# “Shark is the man!”: ethnoknowledge of Brazil’s South Bahia fishermen regarding shark behaviors

**DOI:** 10.1186/1746-4269-10-54

**Published:** 2014-07-03

**Authors:** Márcio Luiz Vargas Barbosa-Filho, Alexandre Schiavetti, Daniela Trigueirinho Alarcon, Eraldo Medeiros Costa-Neto

**Affiliations:** 1 Departamento de Ciências Biológicas (DCB), Programa de Pós-graduação em Zoologia, Universidade Estadual de Santa Cruz, Rodovia Jorge Amado, Km 16, Bairro Salobrinho, Ilhéus, Bahia, Brazil; 2 Departamento de Ciências Agrárias e Ambientais (DCAA), Universidade Estadual de Santa Cruz, Rodovia Jorge Amado, Km 16, Bairro Salobrinho, Ilhéus, Bahia, Brazil; 3 Programa de Doutorado em Desenvolvimento e Meio Ambiente, Universidade Estadual de Santa Cruz, Rodovia Jorge Amado, Km 16, Bairro Salobrinho, Ilhéus, Bahia, Brazil; 4 Departamento de Ciências Biológicas, Universidade Estadual de Feira de Santana, Avenida Transnordestina S/N, Novo Horizonte, Feira de Santana, Bahia, Brazil

**Keywords:** Elasmobranchs, *Rhincodon typus*, Artisanal fishing, Northeast Brazil

## Abstract

**Background:**

Fishermen’s knowledge is a source of indispensable information in decision-making processes related to efforts to stimulate the management and conservation of fishing resources, especially in developing countries. This study analyzed the knowledge of fishermen from three municipal areas of Bahia in northeast Brazil regarding the behavior repertoire of sharks and the possible influence that these perceptions may have on the inclination to preserve these animals. This is a pioneering study on the ethnobiological aspects of elasmobranchs in Brazil.

**Methods:**

Open, semi-structured interviews with shark fishing specialists were conducted between September 2011 and October 2012. The interviews addressed the fishermen’s profile, fishing techniques and knowledge about sharks, focusing on the behaviours exhibited by sharks. The data were analysed with quantitative approach and conducted with the use of descriptive statistical techniques.

**Results:**

Sixty-five fishermen were interviewed. They descend from the rafting subculture of Brazil’s northeast, which has historically been disregarded by public policies addressing the management and conservation of fishing resources. The fishing fleet involved in shark fishing includes rafts, fishing boats and lobster boats equipped with fishing lines, gillnets, longlines and “esperas”. The informers classified sharks’ behaviour repertoire into 19 ethological categories, related especially to feeding, reproduction, and social and migratory behaviours. Because they identify sharks as predators, the detailed recognition of the behaviours exhibited is crucial both for an efficient catch and to avoid accidents. Therefore, this knowledge is doubly adaptive as it contributes to safer, more lucrative fishing. A feeling of respect for sharks predominates, since informers recognize the ecological role of these animals in marine ecosystems, attributing them the status of leader (or “the man”) in the sea.

**Conclusions:**

This work demonstrates the complexity and robustness of artisanal fishermen’s ichthyological knowledge of sharks. Therefore, we suggest that such knowledge should be considered to develop public policies for the control of the fishing activity, as well as to develop and consolidate the National Action Plan for the Conservation of Shark and Ray Species (PAN - Tubarões e Raias).

## Background

The Elasmobranchii subclass comprises cartilaginous fish, with 500 species of sharks described [1]. Due to their numerous adaptive specializations developed over 400 million years [2], sharks stand out as one of the main predators at the top of the food chain in marine environments [3]. As a consequence, they make an extraordinary contribution to the balance of marine ecosystems [4,5], both by controlling the prey population or by exerting evolutionary pressure as they consume old and sick animals [6].

Global initiatives to conserve elasmobranchs are still modest and not very effective when compared to the degree of threat to which the populations of these animals are exposed [7]. On a global scale, fishing stands as the primary threat to sharks, which are caught at an annual rate of between 63 and 273 million individuals [8]. This has contributed to the occurrence of trophic cascades in numerous marine ecosystems around the world [5] and to 20% of elasmobranch species currently falling under some extinction risk status [9].

Faced with a context of severe threats, in 1999, the United Nations Food and Agriculture Organization (FAO) launched the International Plan of Action for the Conservation and Management of Sharks (IPOA–Sharks), a voluntary instrument that applies to all nations where these animals are fished [10]. This Plan establishes a set of activities that participating countries have to fulfil, including the development of a National Action Plan for the Conservation and Management of Sharks (NPOA–Sharks). Although Brazil has not reached this target, in 2012, the Brazilian Society for the Study of Elasmobranchs (SBEEL), with support from the Chico Mendes Institute for Biodiversity Conservation (ICMBio) and the Ministry of Environment (MMA), conducted workshops in the country for the purpose of preparing and debating the National Plan of Action for the Conservation of Elasmobranchs (PAN-Tubarões e Raias).

Eighty-eight shark species [11] have been recorded in the Brazilian coast, of which 12 are threatened with extinction and eight are overexploited or threatened with overexploitation. In spite of being one of the primary sharks biodiversity hotspots in the world [12], historically Brazil has never implemented public policies for the conservation of this group, and this has culminated in fisheries being driven to the point of collapse without any protective measures being taken [13]. However, the decrease in abundance of shark populations resulting from overexploitation causes negative social impacts on fishing communities in Northeast Brazil, as shark meat has been for centuries an important source of protein for these populations [14].

Studies for the conservation of fishing resources are generally based on the biological assessment of stocks. The quantitative methods used for these researches have been developed for application in temperate regions, where industrial fishing exploits a small number of abundant species with long historical series of information available [15]. Contrary to this, artisanal fishing in tropical countries is frequently much more complex: a large variety of fishing gear are used, a large diversity of species are caught that are generally individually scarce in numbers, and numerous landing points and many production chains are employed [16]. Therefore Sparre and Venema [15], highlight that the methods used to assess artisanal fishing stocks must be adequate to situations in which data are limited, and so the use of many sources of quantitative and qualitative information should be maximized, together with the traditional knowledge of fishermen.

The feasibility of preserving fishing resources in Brazil is connected with the need to consolidate a fishing management and study model that takes into consideration both fishing characteristics and human needs [17]. It is clear that Brazilian institutions concerned with research and management of natural environments are faced with the challenge of proposing new conservation alternatives based on an ethnoconservationist model that benefits the maintenance of natural biodiversity and cultural diversity [18].

Marine ethnobiology focuses on studies about the relationships between human societies and the marine Biota of oceanic ecosystems [19]. Native human populations in coastal areas present a wide gamut of knowledge and adaptations qualifying them to survive in these environments [20,21]. It is necessary to study and understand in detail such adaptations and incorporate it into strategies for the coastal areas management, contributing to protect 2.6 billion people who currently depend on marine resources as their main source of protein [22]. To accomplish this incorporation, one possibility is to combine the knowledge of both the policy makers and the natural resources users, such as the fishing communities [23]. According to Johannes, Freeman and Hamilton [24], fishermen can provide scientists with relevant information about the distribution, diet, reproduction, behaviour, abundance and indications of fish overexploitation.

The integration of academic knowledge and fishermen’s knowledge favours a contextualized analysis, connected with the reality of these social actors, which can result in management practices that are more adequate to local fishing resources [23]. It is no surprise that Brazil’s MMA 2008–2011 Pluriannual Plan Assessment Report, in the section assessing the results of and offering future perspectives on the “Sustainable Fishing Resources” Program, strongly highlights the integration of scientific knowledge and traditional fishery knowledge as a means of attaining fishing sustainability in the country in the next few years [25].

This study aims to analyse the fishermen knowledge regarding shark behaviours, as well as their perception concerning the management and conservation of these animals. We believe we could contribute to a better understanding of the human-sharks interactions and, consequently, strengthen recent initiatives for the conservation of these animals in the country.

## Methods

### Study area

The state of Bahia, in the northeast Brazil, has 1,188 kilometres of coastal area, divided into 44 municipal areas containing at least 350 fishing communities [26]. The southern coast of the state is home to some of the most extensive coral formations of the Southwest Atlantic Ocean, the Abrolhos Bank. This fact contributes to the rich diversity of fish species in the Brazilian coast, with more than 250 recorded species [27]. The Abrolhos Bank is located at about 300 kilometres far from the study area and encompasses coral reefs, volcanic islands, shallow banks and channels, covering an area of approximately 6,000 square kilometres. It is an extension of the eastern Brazilian continental shelf, and is quite shallow where the reefs are located [28], less than 30 meters.

In this region, fishing in the reef zones of the continental shelf is an age-old activity of high cultural and economic relevance [29], in spite of being little known or documented [30].

**Figure 1:**
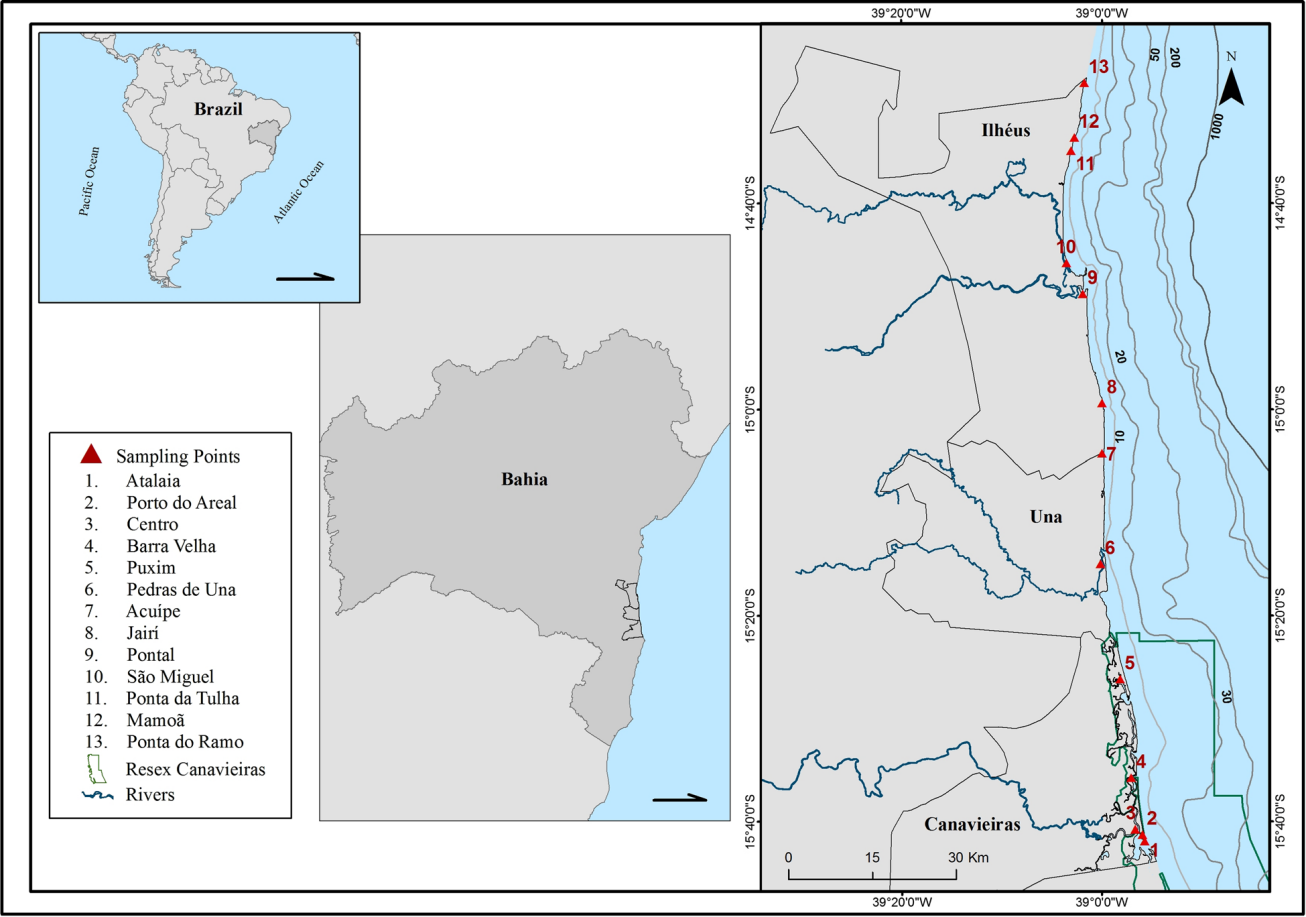
Map of the study area, highlighting the communities where the interviews were conducted.

The study area comprises the municipal areas of Ilhéus, Una and Canavieiras, with an extent of approximately 200 kilometres (14°48′ S, 39°1′W and 15°40′ S, 38°56′ W), within which the Ports of Malhado and Pontal in Ilhéus and Porto Grande in the municipality of Canavieiras stand out for their fishing production [26]. The town of Ilhéus is home to two Fishermen’s Colonies, Z-19 and Z-34, with a total of 6,000 associated fishermen from the area and also from neighbouring towns [31], such as the municipality of Una. Canavieiras Z-20 Fishermen’s Colony has approximately 1,000 associates.Along the study area, there are 13 districts or communities where marine fishermen specialized in shark fishing live (Figure 1).

The Canavieiras Extractive Reserve (CER) is the most important Marine Protection Area (MPA) in the study region. It is a federal MPA that includes the territories of the municipalities of Una, Canavieiras and Belmonte and has a total area of 100,645.85 hectares. The CER is part of the marine and coastal biomes and was implemented on 05 June 2006 [32]. It has an active Executive Board whose members work to approve a management plan and the hiring of a Civil Society Organization of Public Interest (OSCIP), a public interest non-governmental organization, to co-manage it [33]. The CER has had a fishing agreement in force since 2006, establishing rules for the sustainable use of fishing resources. However, this document does not include rules of use for elasmobranchs.

### Data collection

This study was approved by the Committee of Ethics in Research with Human Beings of the State University of Santa Cruz (CEP/UESC 25275) and by the System of Authorization and Information in Biodiversity (SISBIO 33276–1), as a means of complying with an ICMBio requirement (IN 154 de 2007) to conduct scientific studies within federal MPAs, such as the CER.

The first information-collecting phase was conducted using the “data generating methodology” [34], with open-ended interview questions to obtain maximum information and local categories of knowledge and perceptions. This phase was conducted during the months of September and October 2011 via conversations with 14 fishermen during visits to Fishing Colonies Z-34 (n = 8) and Z-20 (n = 6).

Examples of questions asked are “What types of sharks do you know?” and “Why does it have this name?”. This is how we arrived at the species’ scientific names — taking into consideration the “taxonomic clues” criteria, which is when scientific names are obtained by consulting the scientific literature, with a focus on the common names in the areas where the species occur.

Between February and October 2012, semi-structured interviews with open-ended questions were conducted with informers from Canavieiras, Una and Ilhéus. A form was prepared for this purpose, containing general questions regarding fishing in the region and specific aspects involving shark fishing knowledge, such as fishing fleet, the fishing gear involved, fish behaviour repertoire, and the subjective feelings of fishermen arising from their perceptions about different behaviours.

The interviewed fishermen were chosen by selecting “native specialists”, who were those individuals that show some cultural knowledge of the activity, recognized as such by themselves and the community [35] and, in the case of this study, having over 15 years of fishing experience in South Bahia. All interviews were recorded with a digital recorder.

We used the method of checklist interview [36] in order to make the taxonomic correspondence between the names used in the Linnean systematics and those cited by our informers for the shark species caught in the study area. For doing that, interviews were conducted through visual stimulation with a set of 30 printed pictures of different species of shark that inhabit Bahia seashore, using two of them for study control [30,37,38]. Some of the photos used were downloaded from the site *Fishbase*[39] and others from sites with stock images for scientific purposes.

### Data analysis

To record fishermen ichthyological knowledge, we used the model of integrating various individual competencies [Hays *apud*[40], in which all information supplied are taken into account. The quantitative approach was conducted with the use of descriptive statistical techniques.

The controls consisted of tests to verify the consistency and validity of the answers, resorting to repeated interviews in synchronous situations, when the same question was asked to different persons with a short time interval between them [40]. When the same information was repeated by the majority of informers, these were taken as memes [41], which are the shortest verifiable pieces of cultural information, like self-duplicating entities that can be passed on within a certain population by way of speaking [42].

By convention, we considered as the main popular name given to a certain species of shark, the one cited by at least 15% (n = 10) of the informers. The adoption of a relatively small amount of citations is due to the extensive phenotypic similarity among the species of sharks caught by Bahia fishermen and the highly diverse popular names they use to classify these animals [43].

## Results and discussion

### Fishermen and shark fishing profile

Seventy-one male shark fishing specialists were identified; however, five declined to take part in the study and one was not found. Of the 65 interviewed fishermen, 38 live in Canavieiras, 25 in Ilhéus, and two in Una. Eight of the interviewed fishermen are retired.

Their working time in fisheries varied between 30 and 87 years, with a mode and median of 52 and 53 years, respectively. The informers were first initiated into fishing by their fathers, close relatives, or neighbours, and it is common to hear fishermen reporting they started working in marine fishing at 10 years of age. This initiation resulted in school evasion, which in turn can explain the high level of illiteracy (93.9%) among informers.

We found that a large majority (97.1%) of fishermen are native to the coastal communities of Brazil’s northeast and that they descend from the numerous indigenous ethnic groups who have survived for centuries off the natural resources of the Atlantic rainforest and Bahia coast [44]. From an operational point of view, the traditional Brazilian populations may be placed under a number of “cultural areas” or subcultures, with the informers in this study being allocated in Brazil’s northeast rafter subculture [44].

Sharks are regionally called “cação”. All vessels involved in shark fishing are licensed to catch fish, and 13.8% are also licensed for lobster fishing. Their typological profile is as follows: 32.3% are rafts, measuring an average of 7 meters in length; 58.5% are fishing boats, measuring an average of 10.3 meters; and 9.2% are lobster boats, with an average length of 11.1 meters. The primary construction material is wood, present in 95.4% of the vessels; for the construction of the remaining boats is used fiberglass. Even today, the construction of rafts remains as described by Forman [45]. In relation to the propulsion system utilized, 66.2% have a motor, 18.5% are driven by paddle and/or sail, and 15.4% by “stick” – a wooden rod that is about 19 feet long and has a diameter of 12 inches, which is pushed against the seabed.

The autonomy of fishing vessels varies from a few hours to up to 25 days at the sea, according to the typological profile and to the effort towards catching the fish. In relation to fishery areas, a high amplitude was registered in the bathymetry with the depth ranging between 3 and 300 meters, and in the distance from the coast ranging from 0.1 to 70 nautical miles depending on the type of vessel, on the targeted species, and on the climatic and oceanographic conditions the fishing occurs.

Concerning the technological instrumentation, we observed that 27.7% of the boats present no technological devices for geographical displacement, communication or prospection to use during fishing. On the other hand, there are compasses in 58.5% of the vessels, 43.1% have electronic probes, 40% have global positioning system devices (GPS), and 67.7% are equipped with radio communication.

According to the notion of time perceived by the fishermen, there are two seasons: “winter” (April–August), which beginning is marked by the time when “the sea thickens”, and the water gets “dirtier” due to the rains episodes that are common in this period; and “summer” (September–March), when the sea “is calmer” and the water gets “clearer and warmer”. Other studies about Bahia artisanal fishermen have shown a perception of seasonal models similar to those observed in the present study [41,46,47].

The fishermen who participated in the study, separated fish in three different ethnocategories, according to the temporal succession of ethnospecies throughout the year. These are: “winter fish”, “summer fish”, and “fish caught all over the year”, such as previously observed by Mourão and Nordi [48], who studied fishing communities near the estuary of Mamanguape River in Paraíba State, northeast Brazil.

Among shark fishing specialists, we recorded the use of four different types of fishing gear: hand line, gillnet, longline and “esperas”, the latter being the only one made especially to catch this type of fish. However, this gear is not the only that catch sharks in South Bahia, because sharks are also incidentally caught in shrimp trawl nets, cast nets, beach seine nets and fishing rods, both along the coast and offshore. However, the fishermen involved with these types of captures are not specialists in shark fishing.

The hand line is the main fishing gear to catch the “cação”, it is employed in 78.5% of the vessels. There are three types of hand line: “boiêra” with the help of a cork (surface), “bottom” (bottom) or “mid-water” (water column), with two to four lines *per* fisherman and one to three hooks on each line. In fact, the hand line figures the major gear in Bahia and is responsible for a significant part of fish production in the state [49], as it enables the redirection of fishing effort throughout the year, optimizing the exploitation of the species diversity.

Gillnet are used in 55.4% of the vessels, ranging from 3 to 100 nets in each boat. The length of the nets ranges from 50 to 500 meters (100 meters, on average). The height of the nets varies from 2 to 7 meters. The nets may be used on the water surface or near the substratum, and usually are checked by the fishermen once or twice *per* day to verify if any fish was caught.

Six informers reported having recovered lost nets, indicating that these nets are harmful as the fish as the get tangled in the equipment attracting other animals, including sharks which come to the net to eat opportunistically. This continuous capture of fish by forgotten, lost or discarded nets is known as “ghost fishing” and generates significant negative impacts on the ecosystems, affecting stocks of fish and endangering species in coastal and ocean waters [50].

Longlines fishing are employed in 40% of the vessels, and 10.8% of them are equipped with two longlines. In the study area there are two variations of longlines: “surface” and “bottom”. The informers say that in the first case the capture of sharks is less likely, since the hooks are often tied directly to nylon lines. The bottom longline makes feasible the capture of sharks because the hooks are tied to stainless steel cable straps. Regardless the type, the length of the longline may vary from two to six nautical miles and each of the fishing net contains 50 to 1,000 hooks. Respondents informed that the longlines are put on water early in the morning and are collected late in the afternoon. They often complain about foreign boats from other regions of Brazil with large trawl nets exterminate the local fish resources and harm the regional economy.

The “espera” are used by 15.4% of the informers, and one of them is especially prepared to catch sharks. They are composed of a thick cotton rope, and in one end have an iron anchor weighing 100 kilograms, and in the other end a 2-meter-long steel cable is fixed to a stainless steel hook measuring 20 centimetres and weighing one kilogram. There are fishermen in the region using this type of opportunistically fishing to complement the fishing of bony fish. The fishermen interviewed reported that they place the “espera” in the last day to capture shark, since it needs a large quantity of ice for preservation. There are also fishermen that direct their fishing efforts only to sharks, using the “espera”. This type of fishing, despite having always occurred in the region, has increased since 2000, motivated by the high prices of shark fins that according Fong and Anderson [51] are one of the most expensive animal products in the international market.

### Ethology

It is fundamental that fishermen recognize the ethological repertoire of fish species caught, as information of this nature is essential for fishing success [35,44]. In this sense, Cordell [52] states that fishing is not a matter of luck but an understanding of the pattern behaviour of targeted species.

Regionally, in regard to sharks, this understanding is necessary not only for a more efficient catch, but especially as a way to avoid accidents due to the force, speed and violence with which these animals react when they feel imprisoned by the fishing gear. Therefore, it is possible to say that knowledge involving the ethological repertoire of sharks has a doubly adaptive character because it contributes towards more profitable, safer fishing. Marques [35] shows that the connection between fishermen and fish sources may present contradictions and/or ambiguities, since the native fauna can either be a resource or a possibility of risk. In this sense, it is known that the fishermen from the community called Gamboa in Rio de Janeiro (Brazil) have a more accurate perception of the behaviour of sharks, since their interaction with these fish represents a greater risk of accidents [53].

We noted that the interviewed fishermen feel susceptible to shark attacks because of the proximity to sea water while fishing in artisanal vessels, a fact that populates these men’s imaginations, no matter what generation. In fact, sharks have long provoked terror in human beings due to the ferocity and morbidity of some species [54]. Thus, it is noticeable that their behaviour is a component used to construct the classification system of these fish, such as the case of “cação sombreiro”, which is Portuguese for ‘shadow shark’. According to older fishermen, it “waits in the shadow of the hull” and “if you’re not careful, it catches you inside the boat!” In fact, this is the Brazilian denomination for *Isurus oxyrinchus* (Rafinesque, 1810), a species known for jumping out of the water and attacking small vessels [55].

Fishermen regard sharks as having unique characteristics in relation to other captured fish. Their perception of sharks as efficient predators in marine ecosystems culminates in the cultivation not only of feelings of fear by these men but also of respect and admiration for sharks, as they represent the stereotype of successful “command of the area”, of true leaders among marine animals. From the perspective of the Biophilia Hypothesis [56], more precisely the biophilic categorizations of the interactions between humans and natural resources [57], such reverence may be understood as occurring because these fish fall into the aesthetic ideal that natural world’s components can represent.

A curious habit worth noting can be easily identified in the discourse of informers: the use of the expression “the man” (54.4%) instead of saying “the shark”. When questioned about the reasons for referring to sharks exclusively in this manner, fishermen usually argue that “the man is ferocious” and/or “the man is respected”.

With regard to the repertoire of shark behaviours, local fishermen understand that a variety of factors, whether natural (e.g., presence of preferred preys in the environment) or artificial (e.g., when they are attracted by bait discarded along fishing lines), culminate in a diversity of ecological responses from these fish, as they are perfectly adapted to detecting and adequately reacting to the varied stimuli in the environment [58,59]. Therefore, according to native understanding, the behavioural processes of sharks are described and classified into 19 ethological ethnocategories (Table 1), with the main behaviours described related to feeding and reproduction.

**Table 1:** Behavioural repertoire of sharks according to the understanding of informers

**Ethological phenomenon**	**Ethological folk category**	**Sharks involved**
Feeding behaviour	Sharks that are predators	All
	Sharks that eat anything	All
	Sharks that sniff food	All
	Sharks that steal fish	All
	Sharks that cut	Except *Rhincodon typus* (Smith, 1828)
	Sharks that open their mouths and the fish goes in	*Rhincodon typus*
	Sharks that attack from the shadows	*Isurus oxyrinchus*
	Sharks that eat people	*Galeocerdo cuvier*
	Sharks that eat any rubbish	*Galeocerdo cuvier*
Reproductive behaviour	Sharks that give birth to many offspring	All
	Sharks that reproduce when they are still small	All
	Sharks that reproduce nonstop	Some (indefinite)
	Sharks that come to the shore to reproduce	Some (indefinite)
Social behaviour	Sharks that school	Pups in general
	Sharks that live alone	*Rhincodon typus*
Migratory behaviour	Sharks that travel	All
	Sharks that come near in the summer	Most
Sedentary behavior	Sharks that live at certain spots	*Ginglymostoma cirratum* (Bonnaterre, 1788).
Investigative behaviour	Sharks that approach boats	*Rhincodon typus*

### Feeding behaviour

Feeding behaviours are distributed among ten ethological ethnocategories. The most relevant aspect of shark interspecific relations involves their feeding habits [60]. Unfortunately, due to the inherent difficult of studying elasmobranchs in their natural environment, their predatory behaviours are little known by science, especially when compared with those of finfish [61]. Therefore, most knowledge of the group’s feeding ethology comes from anecdotal observations [62].

Local fishermen usually refer to sharks as predators, with the majority (67.7%) reporting that “sharks eat everything”. This meme indicates that sharks are recognized by fishermen as top predators in marine food webs, although in a peculiar manner. The informers usually argue that “sharks are leaders at sea” and/or “all other fish respect sharks”. The fishermen of Guaraqueçaba in the state of Paraná (Brazil) consider sharks as animal species at the top of the food chain [63]. Fishermen living on the Mamanguape River in Paraíba, Brazil also point to this characteristic in sharks; however, to them, this behaviour is related to an essentially opportunistic trophic habit [64].

When questioned regarding whether sharks are important elements of marine environments, 93.8% of informers recognize the relevance of these animals, with quotes from 17% of the informers adequately and consistently confirming the ecological function of sharks in water environments. This sort of argument is exemplified as follows:

*I believe it controls nature too, because it is an exterminator of other fish, isn’t it? It does exterminate them, so it is in control. […] It balances nature.* (E., 58)

This signals a favourable predisposition of fishermen toward preserving these animals, as well as potential success for initiatives to sensitize these social actors to the ecological and social relevance of managing shark populations adequately. In this sense, although the interaction between fishermen and sharks usually involves danger and the risk of financial loss [65], it is possible to identify among the fishermen interviewed positive attitudes and values in relation to these fish; factors that, according to Simpfendorfer et al. [66], can contribute to the success of the participatory management of fish sources.

Fishermen attribute to most sharks the behaviour of “cutting” the prey. This expression is commonly used to designate the feeding habits of fish that tear food apart before swallowing it [67]. The responders make a point of noting how sharp sharks’ teeth are, which according to the interviewed fishermen, are disposed along seven rows.

Another feeding manner described, more precisely for the whale shark (*Rhincodon typus*), is to “open the mouth for the fish to get in”. In their description of the feeding manner of the species, the informers state that these sharks usually “open and close their mouth” and the food “ends up getting in”. This local interpretation of filter-feeding by the species is based on the belief that although they “grow a lot”, whale sharks have a “narrow throat”.

The most relevant species of shark in the region’s fishing culture is the tiger shark (*Galeocerdo cuvier*). This is largely due to its peculiar diet and its strong determination to bite anything that looks like food [56]. Due to this low feeding selectivity, the species is known among Bahia fishermen as a “dustbin with fins” [38]. In the region, the tiger shark is also known as a river mouth shark, as fishermen report that the species inhabits these regions “just waiting for food to come up”. Bigelow and Schroeder [68] have already identified this behaviour to the specie. Therefore, according to the fishermen, the presence of *G. cuvier* at river mouths is related to the species’ diet, as there is more food in these places because of the presence of a number of marine animals that migrate there to reproduce and also because of the dead animals that are carried to the river mouth during the rainy season. There are a number of stories that catch the imagination of local fishermen who recount, with variations, how vessels “capsize at river mouths” and a fisherman falls in the water and is “never seen again”, most likely because he was devoured by a specimen of this species.

*G. cuvier* is also called “jaguara”, a Tupi word meaning jaguar [69]. This denomination has stood the test of time in the collective imagination of various populations of fishermen in northeast Brazil, who retain vestiges of their original indigenous language by attributing Tupi names to ethnospecies of elasmobranchs. These authors also note that apart from the striped pattern analogous to that of jaguars, it is possible that this term has been adopted because of the animal’s ferocity. In summary, one can say that the informers demonstrated detailed knowledge of the feeding behaviour of sharks. Such information is useful because it signals the potential to use fishermen’s knowledge as a source of information on the feeding ecology of these fish.

### Reproductive behaviour

All informers stated that they had caught pregnant females of at least one shark species. This is a strong indication that the region is used by these fish to give birth and to nurse their young. Therefore, we suggest the conduction of studies to confirm this, especially within the CER, because when protected marine areas are managed adequately, they are highly efficient tools for preserving zones that are critical to the lifecycle of elasmobranchs [70].

Apart from the recommendation for such studies, considering that there are still no basic studies on the biology of elasmobranchs in the south of Bahia, it is more urgent to understand the dynamics of local shark populations, because according to the interviewed fishermen, some species are disappearing from fisheries.

One aggravating circumstance is that two of the informers’ perceptions regarding the reproduction of sharks are distorted and could have negative implications for the conservation of the group in the region: that sharks “start reproducing when they are very young”, and that “sharks have many offspring”. The interviewed fishermen reported catching females that although “small” (under five kilograms) were gestating. The main species mentioned were *Rhizoprionodon porosus* (Poey, 1861) and *Rhizoprionodon lalandii* (Valenciennes, 1839). In fact, studies conducted on the Brazilian coast have determined that *R. porosus* and *R. lalandii* females reach maturity when they reach a length of 65 cm [71] and 62 cm [72], respectively. Adding to that, the high frequency with which pregnant females are caught leads to an erroneous generalization that all sharks begin reproducing once they weigh five kilograms, and therefore, catching them is not potentially harmful.

With respect to the fertility of sharks, fishermen’s basis for comparison seems to be the number of children produced by humans at each reproductive event, which is obviously insignificant compared to sharks. Therefore, fishermen do not conceive of the possibility that these animals can become extinct, as seen in this passage:

*I don’t think it will drop (its numbers), because we catch a big shark like this, then it’s 18, 20, sometimes 30. It’s a large quantity of pups* (M., 46).

However, contrary to what the fishermen think, we know that some intrinsic characteristics of elasmobranchs, such as late maturity and low fertility [73], are responsible for the susceptibility of exploited populations to decline [74], as these fish are adapted to the production of a small number of offspring with a high rate of reproductive success [75]. This means that populations have little capability for recovery once they become exhausted [76].

Faced with that little capability and with the difficulty of measuring the impact of artisanal fishing on populations of coastal sharks or sharks that use coastal areas to reproduce and nurse their young [77], we suggest that initiatives be taken to sensitize fishermen, with activities that promote their environmental education, as the observed discrepancies in local knowledge are the result of unfamiliarity with sharks’ reproductive biology. The demystification of fishermen’s beliefs could contribute to a change in the attitude of fishermen to one that is more favourable to the conservation of sharks.

### Social behaviour

A total of 66.2% of informers reported that sharks school at least once in their lifetime. For 35.4% of them, this aggregation would be restricted to the offspring, which commonly “live together”, but when they grow they “become separated”. This information can be ascertained by the frequency with which pups are caught in large numbers by gillnets or shrimp nets at the coast.

The fishermen were naturally predisposed to free the pups caught in the nets because they understand the need to protect sharks in this stage of their lives. However, they report that pups are frequently already dead when caught. Because many shark populations around the world have been plundered by accidental catch in commercial fisheries [65], we suggest that studies be conducted to quantify the impact of indiscriminate, unmonitored catching at many phases of life on the species in the region, as a way of making it feasible to adopt measures aimed at the sustainable exploitation of these resources.

The majority (53.8%) of fishermen pointed that whale shark (*Rhincodon typus*) has a solitary life and only two fishermen reported the sight of two of them together, as it was reported by fishermen from Bajo, Indonesia [78]. In fact, these fish are usually observed alone [79], although aggregations of over 420 individuals have been seen in the Yucatán Peninsula, Mexico, coinciding with a great emergence of zooplankton [80]. Despite recent advances in scientific knowledge on the species, there are still gaps concerning the new-borns life history and those adults that are not usually found in feeding aggregations [81], as it seems to be the case of the specimens observed by the fishermen of the present study.

**Figure 2:**
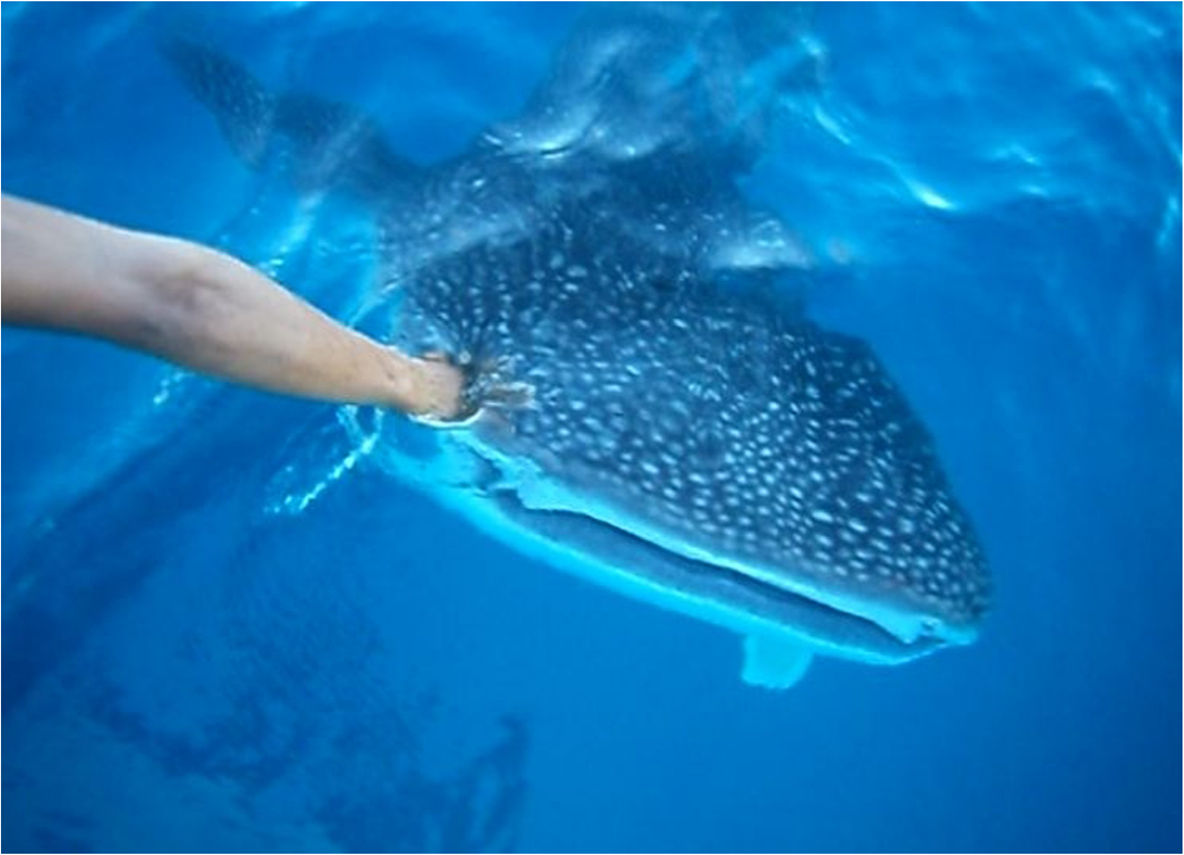
**Fisherman touching the head of a *Rhincodon typus *** **specimen in the south of Bahia, Brazil.**

Because of the inquisitive behaviour exhibited by *R. typus* in the presence of fishing boats, local fishermen often call them “curious”. In these meetings, informers also demonstrate great curiosity, touching them with the hand (Figure 2), hitting the animal head with wooden sticks, or even stabbing them with knives to find out their reaction.

Behavioural studies have focused on the interactions between divers and whale sharks as this practice may affect the behaviour, habitat and ecology of these fish [82]. In the situations in which whale sharks are touched by divers, the ethological answers are very different; some of the whale sharks seem to tolerate human touch [83], and others show signs of stress and respond with various types of defensive behaviour [84]. In fact, it is not possible to state accurately the influence of these kinds of interaction on the animal’s well-being, although it is known that one of the main potential long-term impacts is related to the influence of stress and injuries inflicted by humans [85]. Because of the fact that the species is considered “vulnerable to extinction” by the International Union for Conservation of Nature [86], and the mutual curiosity and the risks involved in the interaction between local fishermen and whale sharks, it is absolutely necessary the implementation of actions with the local fishermen in order to instruct them about what can be done to avoid hazards the animal behaviour when they meeting occurs.

### Migratory behaviour

Among the most important knowledge of Brazilian artisanal fishermen is the seasonable distribution and abundance of the species caught [87]. The informers usually point to sharks as being fish that “walk” or “go walkabout”, which are expressions used to designate migration. However, the fact that “sharks are not only from here”, as highlighted by fishermen, culminates in a distorted belief that on the global level it would be impossible for all sharks to be caught and that shark populations therefore are inexhaustible resources.

According to the time reference perceived by South Bahia fishermen, there are two seasons in the year: “winter” (from April to August), which begins when the “sea thickens” and the water is “dirtier” because of the frequent rain in this period; and “summer” (between September and March), which is when the sea “gets calmer” and the water is “clearer and warmer”. Therefore, according to the perception of the informers, fishing resources are distributed into three different ethnocategories according to the time succession of ethnospecies along the year: “winter fish”, “summer fish” and “year-round fish”.

Most informers (86.2%) stated that there is seasonality to sightings and captures of sharks in South Bahia during the year. Nonetheless, during the “summer” no fishing activities are directed exclusively at this group of fish. Such seasonality is related to the seasonal changes in oceanographic characteristics, including phenological aspects exhibited by the sharks and also changes in marine trophic dynamics among different periods.

Among the informers who reported a seasonal distribution of sharks, most (75%) argued that sharks are present mostly in the summer. Because of the low navigation autonomy of most vessels in South Bahia, which contributes to shark fishing taking place especially along the coast, “summer” is reported as “the time when sharks get close to the coast”. When studying the distribution of fish near the fishermen of Ubatuba in the coast of São Paulo (Brazil), Clauzet et al. [88] noted that sharks only swim near the coast in the summer; however, no reasons were reported in this study for such behaviour.

The main environmental factors determining the seasonality of shark presence in the studied region during the year were variations in the temperature and degree of turbidity of seawater. So, most of the informers said that sharks “appear” in the summer because it is the time of year when the sea water is “warmer” (73.5%) and “cleaner” (78.1%). There is a deficiency of scientific oceanographic data for the study area and, only recently, Eça et al. [89] published the first information on this subject for the coastal region. However, in this study, the authors did not include the temporal dimension of oceanographic variables throughout the year, which makes it impossible a deeper discussion of this seasonal distribution that fishermen perceive to sharks.Fishermen also stated that the females of some shark species “approach the shore” or “river mouths” in the summer to reproduce. The main species identified as behaving in this way were *Carcharhinus limbatus* (Valenciennes, 1841), *Sphyrna lewini* (Griffith & Smith, 1834), *R. lalandii* and *R. porosus*. The fishermen emphasized that these species are caught most frequently, a fact that could be verified during periodic visits to fishing landing points in the region, where the dominant catch were pregnant females (their fetuses are commercialized), newborns and the young of these species. It is probable that the dominance of these species in the catch is a pattern in Brazilian artisanal fishing, as studies conducted in various regions in the country have revealed similar specific compositions [90-92].

Regionally, the argument that these species “approach the shore” is verified by fishermen who operate vessels with coast-restricted autonomy, such as rafts and fishing boats up to nine meters long. These informers also recounted catching pregnant females in the coast at the beginning of the “summer” and that from that time onward, it is common to catch large quantities of offspring. This can be verified in the following passage:

*Summer is the time when sharks are more numerous. They come to the shore to reproduce. Science tells it and it is proved! Most of the sharks that I caught were pregnant. They come from high seas to the Brazilian coast to reproduce* (J., 54).

According Vooren and Klippel [70], females of many shark species whose adults usually live away from the coast migrate to reproduce near the shore, where newborns find more protection and a greater abundance of food.

**Figure 3:**
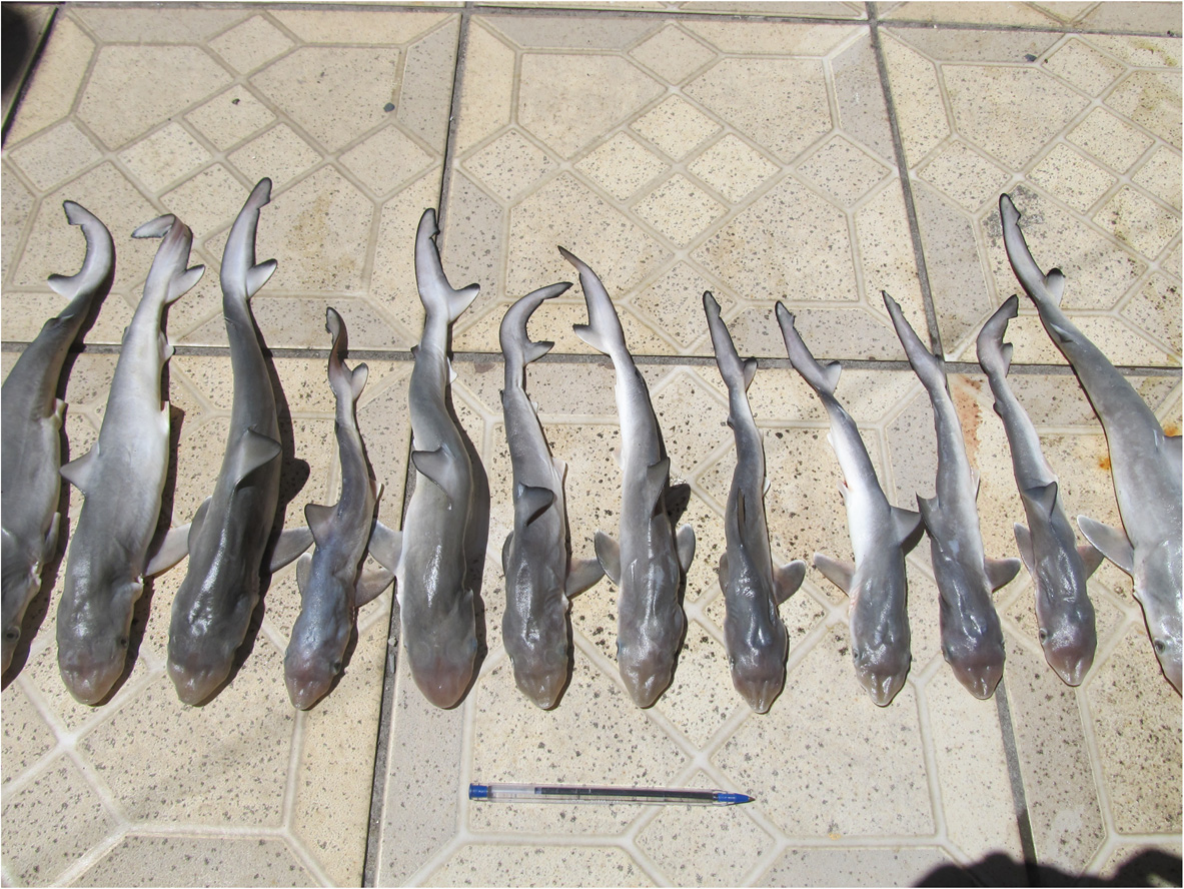
**Newborn *Rhizoprionodon porosus *** **specimens collected at the Ilhéus street market.**

In relation to the commercialization of sharks, *R. porosus* is the species most frequently caught and commercialized by local fishermen. Therefore, it was possible to establish during visits to the Ilhéus street market that during the “summer” months, there is a large offering of newborns, aborted foetuses and those removed from the belly of females for sale. During this season, their price per kilogram is up to US$7.50 for top-quality fish. The high price is due to the local preference for pups, which are an ingredient in “moqueca de caçonete” (baby shark stew), a highly relevant delicacy for the region’s culture and tourism. In this study, a few specimens of the species (Figure 3) were obtained at the Ilhéus market in February 2012, most of them measuring less than 30 centimetres.

According to Sparre and Venema [15], tropical marine fish recruitment patterns are generally not well-understood. These authors also argue that information on the seasonal recruitment patterns of commercial species is a fundamental prerequisite to the application of methods to assess marine fishing stocks. In light of this context and of the potentials threats for the declining in shark populations [93] of Brazil and of other countries in the world [75], informations for the fishing community, as well as the elaboration of folders about the role of cubs of shark “cação” and how the fishermen should proceed when cubs are captured alive should discourage the capture and consequently the local consumption of these fishes. Additionally, because of the growing fishing pressure on coastal elasmobranch populations in Northeast Brazil, information on their biological characteristics and life history is needed [71], as such measures are fundamental for the basis of decisions favourable to the conservation of local stocks by determining acceptable levels of fishing effort.

Due to the lack of biological studies involving elasmobranch populations in the region, it is necessary to conduct scientific research to contribute to better knowledge of the reproductive biology of local population of these fish. Such work, apart from contributing to the conservation of local shark populations, would be a means of analysing the real potential of information imparted by artisanal fishermen to locate elasmobranch nursing areas in the coast of Northeast Brazil, as this knowledge is still very precarious [94].

### Threats to shark populations and possibilities of participatory fishery management

The commercial shark fishing often has a particular dynamic: it begins with a great volume fish, followed by a rapid decrease in the income and the collapse of local populations [4,95,96]. In this sense, the vast majority (90.8%) of the informers from our study reported decreasing in the quantity and in the length in 67.7% of shark specimens caught in the last 15 years. This reduction is usually mentioned with the use of terms such as “rarity” or “difficulty” to capture sharks. Furthermore, the terms “failure” and “flaw” are used to designate the abrupt declines in the fishery incomes, and they seem to be equivalent to ethical expressions used in Biology, such as “collapse of fishing” or “depleted fish stocks”. Unfortunately, little is known about the dynamics and the conservation status of exploited shark populations in Bahia; thus the testimonies of local fishermen represent the only information on the topic.

The cultural-historical analysis of shark catches is a recurring example of the way in which humans have been using several populations of sharks around the world [97,98]. The informers in this study pointed out several reasons related to fishing for the reduction of the amount of sharks, which is perceived at various scales of space. The fishermen interviewed are aware, of the existence of “the fishing of sharks in which you only take the fins”, and the threat it poses to the specimens worldwide [99]. On the other hand, they usually blame other fishermen (regional or from other regions) for the decline in the local catches of sharks, whose are accused of predatory fishing. Thus, they often mentioned more than one reason for the shortage of these fish.

In addition to the understanding of the major threats posed to global shark populations, the fishermen consistently suggest some management options they think necessary for the shark conservation. They are in agreement with recent research developed in order to protect sharks at risk of extinction, as can be seen in Table 2.

**Table 2:** Threats to shark populations and possibilities for conservation suggested by the fishermen interviewed

**Ethical view**	**Emic view (relative frequency) n = 65**	**Possibilities**
*Finning*[100]	“Fishing that only takes shark fins (63,4%)	“Best monitor”; “Give high fine”
Immature Capture [101]	Fish small sharks (61,9%)	“drop the baby sharks”, put closure, “increase the mesh of the nets”
*Bycatch* Capture [102]	“Fishing with trawlnet”, “fishnet excess”, “stuck with longline hook in steel” (46,8%)	“forbid trawling”, “control gillnets use”, “forbid longline hooks stuck with Spiral wire Rope use”
Females Capture [70]	“female fishing”; “fish a female carrying a baby shark in the belly” (15,1%)	“Drop females”; “make closure”

## Conclusions

The knowledge of the fishermen in southern Bahia about sharks derives from centuries of coexistence between them, either by using the same environment or by the exploitation of this fishery resource. Due to the great knowledge and huge cultural background, this knowledge must also be taken into account by policy makers and authorities.

Since the fishermen have refined knowledge on the Biology, the Ecology and the threats regarding sharks, we strongly suggest that this information must be investigated in studies on the population dynamics of species living in the region, especially within the CER, to safeguard local populations through sustainable fishing. Based on that, it will be possible to elaborate management initiatives for the fishing activity and, consequently, for the conservation of these populations.

Studies are also suggested in other regions in order to investigate the social dimension of shark fishing, as a means of consolidating the National Action Plan for the Conservation of Shark and Ray Species. Thus, the knowledge of these social actors, rather than just be considered, should have a central role in the development and adoption of public policies related to participatory management and more suitable for the reality of Brazilian fishing.

## Competing interests

The authors declare that they have no competing interest.

## Authors’ contributions

MLVBF wrote the entire manuscript, prepared the interview form, and worked in the collection, organization and interpretation of the data. AS, DTA and EMCN wrote parts of the manuscript, helped in the preparation of the interview form, contributed ideas to the study, and aided in the discussion and review of the manuscript. All the authors read and approved the final manuscript.
